# Beyond the Silence: COM-B-Informed Provider Barriers to Depression Screening Among Filipino American Patients

**DOI:** 10.3390/healthcare14142166

**Published:** 2026-07-17

**Authors:** Miguel Antonio Fudolig, Andrew Thomas Reyes, Franz Henryk Vergara, Marlon Garzo Saria, Erwin William Leyva, Lorraine S. Evangelista, Reimund Serafica

**Affiliations:** 1 Department of Epidemiology and Biostatistics, School of Public Health, University of Nevada, Las Vegas, NV 89154, USA; miguel.fudolig@unlv.edu; 2 School of Nursing, University of Nevada, Las Vegas, NV 89154, USA; andrewthomas.reyes@unlv.edu; 3 MedStar Harbor Hospital, Baltimore, MD 21225, USA; fvergar1@alumni.jh.edu; 4 Saint John’s Cancer Institute, Providence Saint John’s Health Center, Santa Monica, CA 90404, USA; marlon.saria@outlook.com; 5 College of Nursing, Pharmacy, and Allied Health Sciences, Negros Oriental State University, Dumaguete City 6200, Philippines; erwin.leyva@norsu.edu.ph; 6 Sue & Bill Gross School of Nursing, University of California, Irvine, CA 92697, USA; l.evangelista@uci.edu

**Keywords:** depression screening, Filipino Americans, implementation science, COM-B, primary care, mental health disparities, culturally responsive care

## Abstract

**Background/Objectives:** Routine depression screening is recommended in primary care, yet depression may remain underdetected among Filipino American patients. Provider-level factors, including culturally responsive preparation, communication challenges, workflow constraints, and attitudes toward mental health screening, may influence opportunities for early identification and referral. This exploratory cross-sectional study examined provider perspectives on barriers to depression screening and mental health discussions with Filipino American patients using the Capability, Opportunity, Motivation, and Behavior (COM-B) framework as an implementation-informed organizing lens. **Methods:** A cross-sectional survey was conducted among healthcare providers (N = 81) with experience caring for Filipino American patients in the United States. Survey items assessed provider confidence, perceived adequacy of mental health training, cultural and communication barriers, perceived stigma-related concerns, comfort discussing mental health, and interest in additional culturally responsive resources. Items from the adapted Attitudes Toward Assisting Filipino American Patients with Mental Health Symptoms (ATFA) scale and one item from the Mental Illness Clinicians’ Attitudes scale were conceptually mapped to the COM-B domains. Descriptive statistics, internal consistency estimates, and nonparametric tests were used to summarize findings and explore differences by provider characteristics. **Results:** Most providers recognized that Filipino cultural beliefs and customs may influence mental health help-seeking and symptom expression. Although many providers reported confidence identifying mental health symptoms, fewer reported adequate training to assess mental health concerns among Filipino American patients. Communication barriers, stigma-related concerns, and interest in additional culturally tailored resources were commonly reported. COM-B domain scores were not significantly associated with provider role or years of clinical experience. Providers who identified as Filipino reported greater perceived capability and opportunity related to initiating mental health discussions compared with non-Filipino providers. **Conclusions:** Findings suggest that provider motivation to address mental health concerns may be present, while capability- and opportunity-related barriers, including culturally responsive training, communication support, workflow integration, and referral resources, may remain important targets for future implementation efforts. Because this exploratory study used a modest convenience sample and an adapted measure that requires further psychometric validation, the findings should be interpreted with caution. Larger studies using validated instruments are needed to examine further provider-level determinants of culturally responsive depression screening among Filipino American patients.

## 1. Introduction

Depression is a leading cause of disability worldwide, but is underdiagnosed and undertreated among Filipino American populations [1,2]. Even with the greater awareness of mental health, Filipino Americans may go undetected with depression within the medical care system, and so delay treatment and continue the disparity in mental health [3,4]. Cultural beliefs and communication practices of Filipino American patients may affect their perception and presentation of emotional distress [1]. Cultural norms of emotional control, filial obligations, social harmony, and shame avoidance may inhibit frank discussion of psychiatric distress [5,6]. This can lead to physical symptoms such as fatigue, headaches, sleep problems, or general aches and pains, as well as psychological distress. Routine doctor visits may be less of a setting to catch signs of depression [7].

Although prior research has identified cultural factors such as stigma, somatic symptom presentation, and family-centered values as potentially influencing mental health help-seeking among Filipino Americans [8,9], these characteristics should not be interpreted as universal. Filipino Americans represent a highly heterogeneous population whose experiences vary according to immigration generation, level of acculturation, socioeconomic status, geographic region, healthcare access, language proficiency, and individual life experiences. Accordingly, the cultural factors discussed in this study are presented as potential influences, supported by the literature, rather than as defining characteristics of all Filipino Americans.

Filipino American patients may be a key opportunity to catch depression early in primary care settings, but the screening processes differ [10,11]. Providers are employed in busy clinical settings with competing demands, insufficient time, and a shortage of mental health resources [12,13]. Some providers may also have poor awareness of culturally unique expressions of emotional distress and culturally responsive approaches to mental health assessment [14,15,16]. These limitations highlight the need to further understand provider factors that may influence depression screening methods with Filipino Americans. An implementation-focused framework may help identify the behavioral and contextual influences shaping provider engagement in culturally responsive depression screening practices.

Therefore, the purpose of this study was to examine healthcare providers’ perspectives on depression screening and mental health inquiries among Filipino American patients using the Capability, Opportunity, Motivation, and Behavior (COM-B) framework [15,17]. Specifically, this study examined the roles of provider preparation type, practice experience, and Filipino ethnicity as correlates of practice-related barriers that influence routine mental health discussions in clinical care. Findings may inform culturally grounded interventions to strengthen depression screening practices and improve equitable access to mental health care for Filipino American populations.

### COM-B Framework

Routine depression screening is widely recommended in primary care; however, implementation tends to differ by clinical settings and patient populations, especially in Filipino American patients whose experiences of emotional distress may be shaped by cultural beliefs, stigma, migration experiences, and somatic expressions of symptoms. To better understand provider-related influences on depression screening, this study was guided by the Capability, Opportunity, Motivation, and Behavior (COM-B) framework [15,17,18], which conceptualizes healthcare behaviors as influenced by provider knowledge and skills, environmental and organizational conditions, and individual attitudes and willingness to engage in care practices [17,18]. The COM-B framework has been increasingly adopted in implementation science to identify determinants of mental health behaviors and guide interventions across diverse healthcare settings. Studies applying COM-B have demonstrated its utility in understanding barriers and facilitators to integrating mental health services into primary care, highlighting that providers’ knowledge and clinical skills (Capability), organizational resources and referral systems (Opportunity), and professional confidence and attitudes toward mental health care (Motivation) collectively influence implementation of evidence-based practices [19].

Similarly, COM-B has been used to examine help-seeking behaviors among individuals with schizophrenia, demonstrating that stigma, inadequate mental health literacy, family influences, and limited healthcare access interact across all three behavioral domains to shape engagement with mental health services [20]. Recent applications have further explored mental health literacy and help-seeking among university students [21] provider adherence to smoke-free policies in psychiatric settings [22], perinatal depression screening [23] and cardiometabolic screening among individuals with severe mental illness [24], consistently supporting COM-B as an effective framework for designing multilevel implementation strategies.

Although these studies provide robust evidence for the applicability of COM-B across mental health contexts, relatively few investigations have examined culturally specific determinants of depression screening among Asian American populations. Existing evidence suggests that Asian Americans experience unique barriers, including limited mental health literacy, cultural stigma, language barriers, and reduced access to culturally responsive services [4,25,26,27], which collectively influence capability, opportunity, and motivation to engage in mental health care. Moreover, most studies aggregate Asian Americans into a single racial category, limiting understanding of subgroup-specific experiences despite substantial heterogeneity across communities. In this study, capability reflected providers’ preparation for culturally informed depression assessment; opportunity referred to clinical workflow and organizational resources; and motivation reflected providers’ attitudes and willingness to initiate mental health discussions. The COM-B framework provided a useful approach for identifying practical, potentially modifiable factors influencing culturally responsive depression screening in routine primary care settings [15].

## 2. Materials and Methods

### 2.1. Design and Participants

This exploratory cross-sectional descriptive study examined provider-level perspectives on barriers to depression screening and mental health inquiry among Filipino American patients. Participants included physicians, nurse practitioners, physician assistants, and other licensed healthcare professionals practicing in the United States.

### 2.2. Participant Recruitment and Data Collection

Participants were recruited through professional organizations, healthcare networks, and community partnerships supporting Filipino American populations in the United States. Recruitment materials were circulated electronically via social media outlets and community-based healthcare networks. Because recruitment occurred through open electronic distribution channels, professional networks, social media, and community-based healthcare networks, the total number of individuals who received or viewed the survey invitation could not be determined. Therefore, a formal response rate could not be calculated.

Eligible participants were licensed healthcare providers presently practicing in the United States and providing direct care to adult Filipino American patients in primary care, behavioral health, community health, or outpatient clinical settings. Eligible providers included physicians, nurse practitioners, physician assistants, nurses, and other licensed clinicians involved in patient assessment and care delivery. Participants must be at least 18 years of age, be able to read and understand English, and be currently engaged in clinical practice involving interactions with Filipino American patients.

The exclusion criteria were: not currently practicing in the United States; not providing direct patient care; and no experience caring for Filipino American patients. Also excluded were students, trainees without independent clinical responsibilities, administrative personnel without patient care responsibilities, and retired healthcare providers. Mental health and psychiatry providers were intentionally excluded because the primary objective of this study was to examine provider-level determinants of routine depression screening in non-psychiatric healthcare settings, where the initial identification of depressive symptoms most commonly occurs before referral to specialty mental health services. Eligible participants completed an anonymous online survey administered via Qualtrics. The survey was entirely voluntary, and no personal identifiable information was collected to protect confidentiality and reduce potential response bias. Before participating in the survey, participants reviewed an electronic informed consent statement outlining the study’s purpose, procedures, risks, benefits, and confidentiality protections. The study received exempt approval from the University of Nevada, Las Vegas Institutional Review Board.

### 2.3. Measures

The survey included the Attitudes Toward Assisting Filipino American Patients with Mental Health Symptoms (ATFA) scale, a study-specific measure developed to assess provider perceptions of culturally relevant barriers to depression screening and mental health discussions with Filipino American patients. The ATFA items assessed provider confidence, perceived adequacy of training, communication barriers, stigma-related concerns, cultural beliefs that influence symptom expression, comfort with discussing mental health, and interest in additional culturally responsive resources. The ATFA scale consisted of seven items with 5-point Likert-scale responses ranging from 1 (Strongly Agree) to 5 (Strongly Disagree). Higher scores indicated lower perceived cultural awareness, confidence, or readiness related to mental health discussions with Filipino American patients.

The study also included an item from the Mental Illness Clinicians’ Attitudes (MICA) scale to assess providers’ attitudes toward mental illness and mental health care. The MICA scale [28,29] is a widely used, validated instrument developed to assess stigmatizing attitudes among healthcare professionals working with individuals experiencing mental illness. Previous studies have demonstrated acceptable psychometric properties for the MICA scale, including good internal consistency reliability with reported Cronbach’s alpha coefficients generally ranging from 0.72 to 0.79 across healthcare provider populations [28,29,30]. The measure showed good construct validity and has been used extensively in studies of physician stigma, mental health training, and providers’ attitudes toward psychiatric care in multidisciplinary healthcare settings (18). The MICA scale was used to systematically assess providers’ attitudes toward mental illness, stigma, and engagement in mental health care practices, thereby increasing measurement rigor in this study. MICA items used a 6-item Likert scale (1: Strongly Agree–6: Strongly Disagree), with higher scores indicating more negative attitudes towards mental illness.

The ATFA was developed for this exploratory study because no existing validated instrument was identified that specifically assessed provider perceptions of culturally relevant barriers to depression screening among Filipino American patients. To support face and content validity, the items were reviewed by an expert panel that included psychiatric mental health nurse practitioners, nurse researchers, and experts in mental health and cultural health disparities [31,32,33]. The scale was also pilot-reviewed to improve clarity, readability, comprehensibility, and cultural appropriateness before administration. However, the ATFA has not yet undergone full psychometric validation, including exploratory or confirmatory factor analysis, test–retest reliability assessment, or validation in a larger and more diverse provider sample.

ATFA items and one MICA item were conceptually mapped to the COM-B framework to organize provider-level determinants of depression screening and mental health discussions with Filipino American patients. The items corresponding to capability, opportunity, and motivation are shown in Table 1. Capability was represented by items assessing provider confidence and perceived adequacy of training to identify or assess mental health concerns. Opportunity was represented by items assessing communication barriers, stigma-related concerns, and interest in additional culturally responsive resources. Motivation was represented by items assessing perceived cultural influences on symptom expression, comfort discussing mental health, and comfort interacting with individuals experiencing mental illness. This mapping was used as an implementation-informed organizing approach rather than as a formal validation of COM-B constructs.

### 2.4. Data Analysis

Descriptive statistics were used to summarize participant demographic characteristics, professional backgrounds, clinical practice characteristics, and survey responses. Frequencies and percentages were computed to describe provider demographics, years of clinical experience, practice settings, and prior experience with Filipino American patients. All data were screened for completeness, consistency, and validity before analysis. Survey responses were screened to eliminate duplicates, incomplete responses, and inappropriate responses. Participants who did not complete the questionnaire or had a large amount of missing data were excluded from the final analysis.

Qualtrics data were screened for data entry errors, inconsistent responses, and out-of-range values. The pattern and extent of missing data were analyzed. Only fully or properly completed questionnaires were included in the final analytic sample. Descriptive results following data cleaning were evaluated using the COM-B framework to identify provider-related factors associated with routine depression screening and mental health inquiry among Filipino American patients. The reliability of the COM-B scale was measured using Cronbach’s alpha (α). Values higher than 0.60 indicate good internal consistency for these measures [33]. The 95% confidence intervals for the α values were estimated using bootstrapping methods through the *psych* package in R [34].

Individual item responses were reported as frequencies and percentages based on whether participants responded affirmatively to each item (Somewhat Agree/Agree/Strongly Agree). Sum scores for each COM-B construct were calculated and reported as medians and quartiles. These sum scores were assessed for associations between provider role, ethnicity, and years of service. Provider roles and experience were aggregated to account for small cell counts. Associations involving ATFA individual items within the COM-B framework were analyzed using the Kruskal–Wallis test. The data analysis was implemented using R statistical software version 4.6.0 [35]. Given the exploratory nature of the study and the modest sample size, inferential analyses were limited to nonparametric methods.

Because the study was exploratory and the final analytic sample was modest (N = 81), an a priori power analysis was not conducted, and the study was not designed to support multivariable modeling or formal psychometric validation of the ATFA scale. Exploratory or confirmatory factor analysis was not performed because the sample size was insufficient to provide a stable estimate of factor structure. Therefore, findings based on the ATFA and COM-B domain scores should be interpreted as preliminary and hypothesis-generating.

## 3. Results

### 3.1. Participant Characteristics

A total of 81 healthcare providers were included in the final analytic sample. As shown in Table 2, physicians represented the largest proportion of participants (58.0%, *n* = 47), followed by nurse practitioners (29.6%, *n* = 24). Most participants reported more than 10 years of clinical experience (77.8%, *n* = 63). The majority identified as Asian (76.5%, *n* = 62), and nearly all participants reported prior experience caring for Filipino American patients (98.8%, *n* = 80). Filipino ethnicity was strongly represented within the sample (67.9%, *n* = 55), reflecting the study’s recruitment through Filipino-serving professional and community networks. Percentages may not total 100% due to rounding.

### 3.2. Reliability

**Figure 1:**
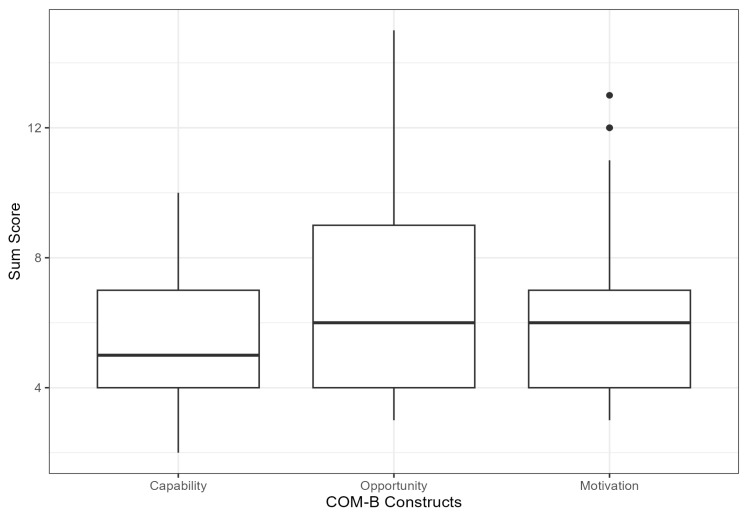
The distribution of the overall sum scores for the capability, opportunity, and motivation constructs of the COM-B framework. The respective ranges of possible scores for the capability, opportunity, and motivation constructs are 2–10, 3–15, and 3–16.

The COM-B-informed item set demonstrated acceptable internal consistency in the current exploratory sample (α = 0.79, 95% CI: 0.52–0.86). Internal consistency estimates were also acceptable for the Capability (α = 0.73, 95% CI: 0.51–0.87), Opportunity (α = 0.72, 95% CI: 0.57–0.82), and Motivation (α = 0.66, 95% CI: 0.51–0.77) domains. These estimates supported preliminary descriptive analysis of domain sum scores in this sample; however, they do not establish construct validity or confirm the factor structure of the ATFA scale. Figure 1 shows the distribution of scores for each construct among participants.

### 3.3. Capability: Training, Cultural Awareness, and Clinical Readiness

Providers generally demonstrated awareness of cultural influences on mental health discussions among Filipino American patients, as evidenced by a median Capability subscale sum score of 5 (Q1–Q3: 4–7). 63% (*n* = 51) of the participants reported that they were confident in identifying mental health symptoms in Filipino patients, but only 42% (*n* = 34) reported receiving adequate training to address these concerns.

**Figure 2:**
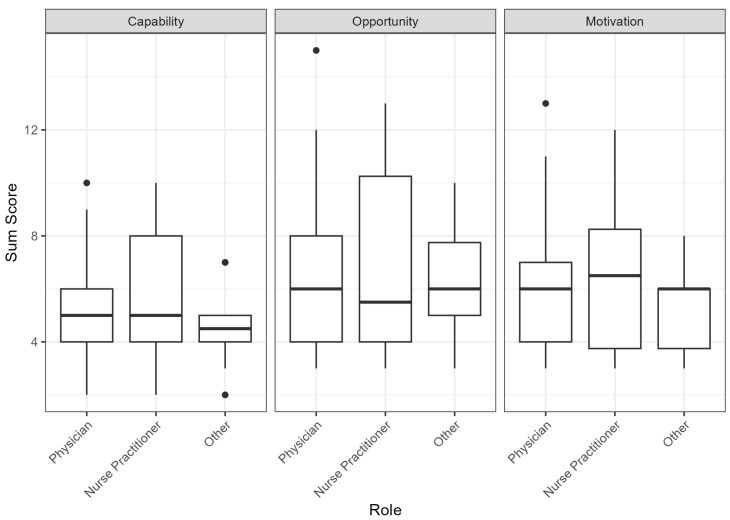
The distribution of overall sum scores for the COM-B framework, stratified by provider role. The respective ranges of possible scores of the capability, opportunity, and motivation constructs are 2–10, 3–15, and 3–16.

**Figure 3:**
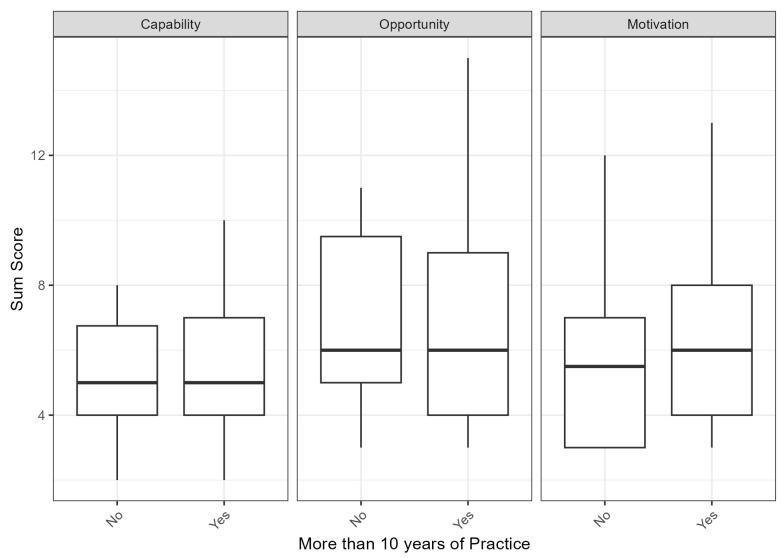
The distribution of overall sum scores for the COM-B framework, stratified by years of experience. The respective ranges of possible scores of the capability, opportunity, and motivation constructs are 2–10, 3–15, and 3–16.

**Figure 4:**
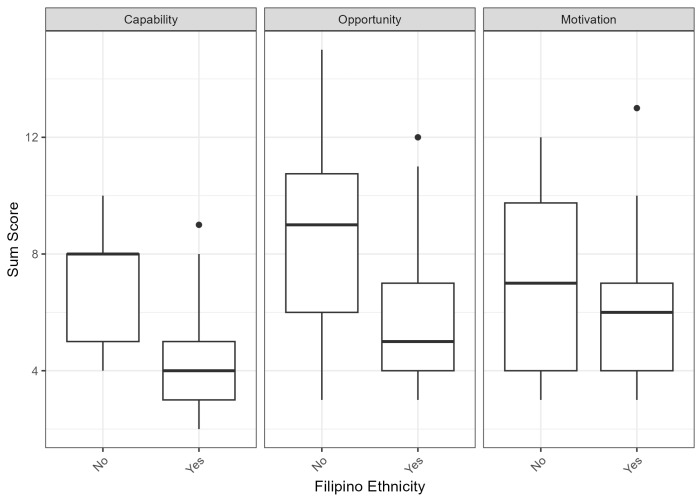
The distribution of overall sum scores for the COM-B framework, stratified by Filipino ethnicity. The respective ranges of possible scores of the capability, opportunity, and motivation constructs are 2–10, 3–15, and 3–16.

There was no evidence of a difference in score distribution between provider roles ($\chi_{2}^{2}$ = 0.98, *p* = 0.61) and years of experience ($\chi_{1}^{2}$ = 0.21, *p* = 0.65). However, Filipino providers recorded a median score of 4 (Q1–Q3: 3–5), while those who were not Filipino recorded a median score of 8 (Q1–Q3: 5–8), indicating that the Filipino providers perceived themselves as more capable in initiating mental health discussions with Filipino American patients ($\chi_{1}^{2}$ = 20.6, *p* < 0.0001). The side-by-side comparisons of these distributions are shown in Figure 2, Figure 3 and Figure 4.

### 3.4. Opportunity: Communication and Workflow Barriers

Communication and workflow-related barriers emerged as important determinants of implementation influencing mental health discussions. The participants recorded a median score of 6 out of 15, indicating recognition of these barriers. Most participants recognized cultural barriers such as stigma surrounding mental health in the community (86.4%, *n* = 70) and communication issues when communicating mental health concepts (56.8%, *n* = 46). 71.6% (*n* = 58) expressed interest in additional culturally tailored resources to address mental health needs in the Filipino community, suggesting that participants were willing to improve their skills to help overcome challenges in initiating conversations about mental health.

Similar to the Capability construct, the provider type ($\chi_{2}^{2}$ = 0.18, *p* = 0.92) and years of experience ($\chi_{1}^{2}$ = 0.18, *p* = 0.67) were not associated with the perception of the existence of these barriers related to the opportunity to initiate mental health conversations and encourage depression screening in the Filipino community (Figure 2 and Figure 3). Filipino providers were more likely to address these opportunities (Median: 5, Q1–Q3: 4–7) compared to non-Filipino providers (Median: 9, Q1–Q3: 6–11), as supported by the results of the Kruskal–Wallis test ($\chi_{1}^{2}$ = 18.1, *p* < 0.0001). These differences were visualized in Figure 2, Figure 3 and Figure 4.

### 3.5. Motivation: Attitudes Toward Mental Health Screening

Findings regarding provider motivation showed generally positive attitudes toward mental health screening, despite ongoing uncertainty about provider roles and current health-seeking behavior. Eighty-five percent (*n* = 69) reported cultural beliefs influence symptom presentation in Filipino patients. 80% (*n* = 65) reported they were comfortable addressing mental illnesses as much as physical illnesses, but only 63% (*n* = 51) reported discussing mental health issues with Filipino patients.

As with the other two constructs, there was no evidence of a difference in attitudes towards factors related to the motivation for depression screening between provider roles (chi-squared = 0.89, *p* = 0.64) and years of experience (chi-squared = 1.59, *p* = 0.21). There was a discernible difference between Filipino (Median: 6, Q1–Q3: 4–7) and non-Filipino providers (Median: 7, Q1–Q3: 4–10), yet it was notably smaller compared to the other two constructs ($\chi_{1}^{2}$ = 4.41, *p* = 0.04).

### 3.6. COM-B Integration of Implementation Determinants

**Figure 5:**
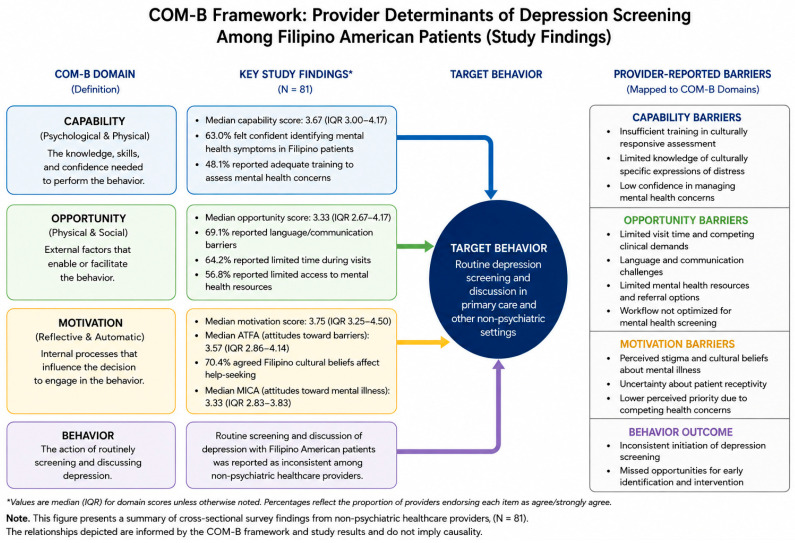
COM-B framework of provider determinants influencing depression screening among Filipino American patients.

The overall findings are conceptually summarized in Figure 5 and operationally mapped in Table 3 using the COM-B framework. Capability-related barriers primarily reflected limited culturally responsive training and uncertainty regarding culturally sensitive mental health discussions. Opportunity-related barriers involved communication difficulties, workflow limitations, and insufficient behavioral health resources. In contrast, providers generally demonstrated strong motivation to engage in mental health screening practices and expressed willingness to participate in additional culturally responsive training and implementation supports. Together, these findings suggest that improving provider capability and opportunity may represent the most practical targets for strengthening culturally responsive depression screening among Filipino American patients.

## 4. Discussion

This exploratory study used the COM-B framework to organize provider-level perspectives on depression screening and mental health inquiry among Filipino American patients. The findings should be interpreted with caution given the modest sample size, nonprobability sampling, high representation of Filipino providers, and the use of a study-specific ATFA scale that requires further psychometric validation. Within these limitations, the study provides preliminary implementation-focused evidence suggesting that providers may be motivated to address mental health concerns. At the same time, capability- and opportunity-related barriers—such as culturally responsive training, communication support, workflow integration, and referral resources—may be important targets for future intervention development.

In the capability domain, many providers recognized the importance of addressing mental health concerns and acknowledged that Filipino cultural norms may shape how emotional distress is expressed and discussed. However, fewer providers reported adequate training to assess mental health concerns among Filipino American patients, suggesting a potential gap between general comfort discussing mental health and preparation for culturally responsive depression screening. Providers who identified as Filipino reported higher perceived capability than non-Filipino providers, which may reflect greater cultural familiarity or lived experience; however, this finding should be interpreted cautiously given the sample composition and exploratory design. These findings are clinically relevant because some Filipino American patients may express emotional distress through physical symptoms, fatigue, sleep disturbance, or stress-related concerns rather than directly disclosing sadness or depression. Without culturally responsive training, providers may miss opportunities for early identification of depression when emotional distress is presented indirectly or somatically [36,37].

In the opportunity domain, providers identified communication, workflow, and referral-related barriers that may affect routine depression screening. More than half of participants reported language or communication barriers when discussing mental health concerns with Filipino American patients. These barriers may extend beyond language alone and may include indirect communication patterns, reluctance to disclose emotional distress, concerns about family burden, or efforts to preserve interpersonal harmony. Filipino providers reported greater perceived opportunity than non-Filipino providers, which may reflect greater familiarity with culturally shaped communication patterns; however, this interpretation should be considered preliminary. Providers also noted practice-level constraints, including limited time during visits, competing clinical demands, limited integration of behavioral health services, and uncertainty about referral pathways. These findings suggest that improving culturally responsive screening may require not only provider education, but also workflow supports, referral resources, and organizational structures that make mental health inquiry easier to incorporate into routine care.

In the motivation domain, providers generally endorsed the importance of addressing mental health concerns and integrating depression screening into routine clinical care. Differences in motivation scores across provider role, years of experience, and Filipino ethnicity were smaller than those observed for capability and opportunity, suggesting that motivation may not be the primary implementation barrier in this sample. Instead, providers appeared generally willing to engage in mental health discussions but identified challenges related to culturally responsive preparation, communication, workflow integration, and referral resources. These findings suggest that future interventions may be most useful if they strengthen providers’ capabilities and opportunities through culturally responsive communication training, practical screening tools, clearer referral pathways, and workflow supports that normalize mental health inquiry during routine encounters.

These findings also have implications for health equity among Filipino American patients. Filipino Americans are often grouped within broader Asian American categories despite important differences in migration history, language use, acculturation, socioeconomic circumstances, cultural beliefs, and mental health stigma [3]. Such aggregation may obscure subgroup-specific barriers to depression recognition and mental health care. In addition, the “model minority” stereotype may contribute to the under-recognition of emotional distress by reinforcing assumptions that mental health concerns are less prevalent or less urgent within Filipino American communities [38].

Primary care and other non-psychiatric healthcare providers may be well positioned to reduce these inequities through culturally responsive communication, earlier recognition of distress, and normalization of mental health discussions during routine encounters. Although exploratory, this study contributes to the implementation science literature by identifying provider-level factors that may influence culturally responsive depression screening among an underrepresented population.

By applying the COM-B framework, this study offers a preliminary implementation-focused lens for understanding provider-level factors that may shape depression screening and mental health discussions with Filipino American patients [39]. Rather than suggesting that provider motivation alone is insufficient, the findings point to capability- and opportunity-related targets that may be addressed through future interventions. Additional studies are needed to evaluate whether culturally responsive provider training, integrated behavioral health workflows, and community-engaged implementation strategies improve depression screening, referral processes, and mental health outcomes in Filipino American communities.

### Practice and Policy Implications

The findings of this exploratory study suggest several potential implications for primary care practice and provider education. Given the modest sample size, nonprobability sampling, high proportion of Filipino providers, and use of a study-specific ATFA scale requiring further psychometric validation, these implications should be interpreted as preliminary rather than definitive.

Participants identified challenges related to culturally responsive communication, perceived adequacy of mental health training, workflow integration, referral processes, and access to culturally appropriate resources. These findings suggest that healthcare organizations may benefit from exploring educational and organizational strategies that support providers in addressing mental health concerns during routine primary care encounters. Potential approaches include culturally responsive communication training, enhanced collaboration with behavioral health professionals, improved referral pathways, and greater availability of culturally appropriate educational and screening resources. Future implementation studies are needed to determine whether these strategies improve provider confidence, screening consistency, referral completion, and patient outcomes.

The findings also reinforce the importance of considering health equity when addressing mental health among Filipino American patients. Broad assumptions about Asian American populations may obscure subgroup-specific patterns of emotional distress, help-seeking, and engagement with mental health care. Primary care and other non-psychiatric providers may be well positioned to support earlier recognition of depression through culturally responsive communication and appropriate referral when clinically indicated.

Although routine depression screening is recommended for adults by the U.S. Preventive Services Task Force, this study was not designed to evaluate healthcare policy, screening effectiveness, or system-level interventions. Therefore, broader policy implications should be interpreted as areas for future investigation rather than direct recommendations from the present findings. The observed patterns related to provider confidence, perceived training, communication barriers, and culturally responsive screening needs provide preliminary evidence that may inform future implementation research. Larger and more representative studies using validated instruments are needed to determine whether organizational supports, workforce development initiatives, culturally responsive implementation strategies, or referral and reimbursement structures improve depression screening and engagement in mental health care among Filipino American populations.

## 5. Limitations

Several limitations should be considered when interpreting the findings. First, the cross-sectional design limits causal inference, and the use of self-reported survey data may have introduced recall or social desirability bias. Second, participants may have had greater interest in mental health, Filipino American health inequities, or culturally responsive care than providers who did not participate. Third, the modest sample size limited subgroup analyses by provider discipline, geographic region, and practice setting. The study also did not include patient perspectives or direct observation of clinical encounters; therefore, the findings reflect provider-reported perceptions rather than observed depression-screening practices. The study did not adequately analyze disparities in immigrant histories, acculturation, language preferences, and socioeconomic circumstances among Filipino American populations. Finally, although the COM-B framework provided a useful lens for thinking about provider behavior, the study did not test specific implementation options.

Because recruitment occurred through open electronic distribution channels, professional networks, social media, and community-based healthcare networks, the total number of individuals who received or viewed the survey invitation could not be determined; therefore, a formal response rate could not be calculated. This limits interpretation of participation patterns and may have introduced selection bias. In addition, the high proportion of Filipino providers in the sample likely reflects recruitment through Filipino-serving networks. It may limit generalizability to non-Filipino providers or providers with less direct experience caring for Filipino American patients.

The validation, test–retest reliability, and factor structure of the COM-B questionnaire have not been tested due to the small sample size. Further refinement of this survey instrument could be explored to address other aspects of perceptions of capability, opportunity, and motivation. Additional study is also needed to evaluate the effectiveness of culturally adapted trainings, integrated behavioral health models, and community-engaged approaches to improve depression screening and mental health outcomes among Filipino American populations.

The adapted ATFA scale was developed to address a gap in culturally specific measures relevant to provider perceptions of depression screening among Filipino American patients. Although the instrument underwent expert and pilot reviews and an internal consistency assessment, it has not undergone full psychometric validation. Exploratory or confirmatory factor analysis, test–retest reliability assessment, and construct validation in a larger and more diverse provider sample were beyond the scope of this exploratory study. Therefore, ATFA-based findings should be interpreted as preliminary and hypothesis-generating. Future research should further evaluate the instrument’s factor structure, construct validity, reliability, and performance across diverse healthcare provider groups.

Finally, although this study focused on Filipino American patients, we acknowledge the substantial heterogeneity within this population. Differences in immigration history, acculturation, socioeconomic circumstances, language use, and regional backgrounds were not measured and therefore could not be examined. Future studies should investigate how these within-group differences influence provider perceptions and depression screening practices.

## 6. Conclusions

This exploratory study used the COM-B framework to examine provider-level perspectives on barriers to depression screening and mental health inquiry among Filipino American patients. Findings suggest that providers may be generally motivated to address mental health concerns. At the same time, capability- and opportunity-related barriers, including culturally responsive training, communication support, workflow integration, and referral resources, may warrant further investigation as targets for implementation strategies. Because the study used a modest convenience sample and a study-specific ATFA scale that requires further psychometric validation, the findings should be interpreted as preliminary. Larger studies using validated instruments, more diverse provider samples, and stronger analytic designs are needed to evaluate provider-level determinants of culturally responsive depression screening and referral practices among Filipino American patients.

## Figures and Tables

**Table 1:** Survey items corresponding to each COM-B construct.

COM-B Construct	Survey Item	Scale	Score Range
Capability	I feel confident identifying mental health symptoms in Filipino patients	ATFA	1–5
Capability	I have received adequate training to assess mental health concerns in Filipino patients.	ATFA	1–5
Opportunity	Language or communication barriers make it more difficult to address mental health concerns in Filipino patients.	ATFA	1–5
Opportunity	I believe stigma around mental illness affects Filipino patients’ willingness to seek help.	ATFA	1–5
Opportunity	I want additional resources or training to better support Filipino patients with mental health needs.	ATFA	1–5
Motivation	I believe cultural beliefs of Filipino patients influence how they express mental health symptoms.	ATFA	1–5
Motivation	I feel comfortable discussing mental health topics with Filipino patients.	ATFA	1–5
Motivation	I feel as comfortable talking to a person with a mental illness as I do talking to a person with a physical illness.	MICA	1–6

**Table 2:** Demographic characteristics.

Characteristic	*n*	%
**Provider Type**		
Physician	47	58.0
Nurse Practitioner	24	29.6
Physician Assistant	2	2.5
Clinical Nurse Specialist	1	1.2
Hospitalist	2	2.5
Specialist	2	2.5
Clinical Nurse Educator	1	1.2
Other/Not Specified	2	2.5
**Years in Practice**		
Less than 1 year	2	2.5
1–5 years	10	12.3
6–10 years	6	7.4
More than 10 years	63	77.8
**Race/Ethnicity**		
Asian	62	76.5
White	14	17.3
Black	2	2.5
Middle Eastern/North African	1	1.2
Asian-Mixed Race	2	2.5
Asian-Filipino Ethnicity	55	67.9
**Prior Experience Caring for Filipino Patients**		
Yes	80	98.8
Not Sure	1	1.2

**Table 3:** COM-B mapping of implementation determinants influencing depression screening.

COM-B Domain	Related Findings	Potential Implementation Strategies
**Capability**	Providers reported confidence in culturally responsive training and in identifying mental health issues, but high uncertainty in initiating culturally sensitive mental health discussions with Filipino American patients.	Culturally informed provider education, communication training, and depression screening workshops focused on somatic symptom presentation and stigma-sensitive interviewing.
**Opportunity**	Providers identified communication barriers and limited culturally specific training within primary care settings.	Integration of culturally tailored screening prompts, interpreter-supported materials, behavioral health referral pathways, and workflow-based screening supports.
**Motivation**	Providers generally valued mental health screening as much as physical checkups and demonstrated willingness to address depression among Filipino American patients despite culturally influenced implementation barriers.	Reinforcement and normalization of the importance of mental health screening through organizational support, continuing education, and community engagement.

*Note.* COM-B = Capability, Opportunity, Motivation, and Behavior framework.

## Data Availability

The data presented in this study are available on request from the corresponding author. The data are not publicly available due to privacy and ethical restrictions related to the protection of participant confidentiality and the potentially identifiable nature of the responses.
